# Multi-Agent System for Intelligent Urban Traffic Management Using Wireless Sensor Networks Data

**DOI:** 10.3390/s22010208

**Published:** 2021-12-29

**Authors:** Maria Viorela Muntean

**Affiliations:** Department of Informatics, Mathematics and Electronics, 1 Decembrie 1918, University of Alba Iulia, 510009 Alba Iulia, Romania; mmuntean@uab.ro

**Keywords:** multi-agent system, classification, forecasting, smart cities, urban traffic management

## Abstract

Intelligent traffic management is an important issue for smart cities. City councils try to implement the newest techniques and performant technologies in order to avoid traffic congestion, to optimize the use of traffic lights, to efficiently use car parking, etc. To find the best solution to this problem, Birmingham City Council decided to allow open-source predictive traffic forecasting by making the real-time datasets available. This paper proposes a multi-agent system (MAS) approach for intelligent urban traffic management in Birmingham using forecasting and classification techniques. The designed agents have the following tasks: forecast the occupancy rates for traffic flow, road junctions and car parking; classify the faults; control and monitor the entire process. The experimental results show that k-nearest neighbor forecasts with high accuracy rates for the traffic data and decision trees build the most accurate model for classifying the faults for their detection and repair in the shortest possible time. The whole learning process is coordinated by a monitoring agent in order to automate Birmingham city’s traffic management.

## 1. Introduction

Nowadays, intelligent data flow management has attracted a large amount of attention in the smart cities domain. Learning the large amount of data collected with smart city infrastructure and technologies has become an important issue as it is necessary in order to make the best decisions in the minimum amount of time.

In this paper, a multi-agent system (MAS) is proposed in order to automate the urban traffic management and control (UTMC) from Birmingham and the West Midlands councils. The intelligent agents embedded forecasting or classification techniques in order to extract knowledge from a large amount of data collected from the sensors.

The proposed architecture is designed to deal with real-time data and to notify a human expert if any anomalies appear regarding traffic flow occupancy rates, road junction occupancy rates and car parking occupancy rates in Birmingham city. It also detects the faults that can appear in the collection equipment and sends real-time notifications.

In the context of smart cities, wireless sensor networks play an important role in developing smart applications [1], emergency applications [2], seismic monitoring buildings [3], air pollution monitoring [4], characterization of urban anomalous noise events [5], and many others.

Regarding intelligent monitoring applications, Byeongjoon Noh et al. [6] propose data mining techniques for reducing road crossing accidents in Osan city, South Korea, by clustering different types of vehicle and pedestrian interactions. The authors used object detection by applying the regional convolutional neural network model on image frames collected by a video camera. Using feature extraction, cluster analysis, decision trees and rule models, the authors presented useful information and knowledge to the decision maker.

The approach proposed in [7] uses decision support systems for disaster management in smart cities. The authors collected and prepared past event data, weather data, sensor data and satellite data and constructed models based on convolutional neural networks, generative adversarial network and reinforcement learning to accurately predict wildfire direction. Experimental results show that the proposed framework is suitable for automatic fire detection and can assist in forecasting disasters.

Car park occupancy detection rates are also an important issue in smart cities. Lun-Chi Chen et al. [8] propose a smart control system that uses camera images and videos to control streetlights, detect vehicles and calculate the occupancy rates using a voting mechanism.

Traffic noise prediction using a recurrent neural network was proposed by Xue Zhang et al. [9]. The authors studied the traffic noise in Blansko, Czech Republic, using video recording and audio recording. The audio and video data preprocessing techniques help the model to better distinguish between the dataset classes.

For pedestrian traffic light classification, in [10], computer vision technology and transfer learning models are used. Big image data for road traffic is analyzed in [11] using different topologies of convolutional neural networks.

In [12], an urban transport multi-media data approach for resilience management in smart cities is described. In [13], a smart decision support system is proposed to support decision-making processes in a smart city environment.

Multi-agent systems (MAS) are used in smart city application in order to automate and monitor different processes. A guidance system for route recommendations for travelers in Nottingham and Sofia is described in [14]. The authors propose a multi-agent system composed of the following: a managing agent, transport agents, a traffic data fetcher, a commuter agent, a route recommender agent and a visualization agent. A multi-agent recommender system for the tourism sector in the Alba Iulia smart city, Romania, is proposed in [15].

Billhardt et al. [16] show that the coordination in smart cities can be implemented through multi-agent systems. They propose an architecture consisting of user agents and smart building agents and test the system for several real-world applications.

David Eneko Ruiz de Gauna et al. [17] propose an approach that combines electric vehicles with the advantages of multi-agent systems in order to solve the problems of pollution and congestion. Davide Andrea Guastella et al. [18] designed a cooperative multi-agent system to reduce the number of necessary sensors to be deployed in a smart city.

Due to the great number of vehicles and limited space, the city councils have to solve problems such as traffic congestion or crowded parking. Additionally, efficient use of traffic lights is a main concern. The current studies propose different solutions based on methods ranging from video and image processing to extracting knowledge from large amounts of data.

Short-term traffic flow prediction for the I-64 in St. Louis, Missouri [19] is a real-world case that shows that machine learning methods are efficient techniques for analyzing such data. Traffic prediction on real-world traffic datasets was also performed using the spatio-temporal attention mechanism-based dynamic network model that learns dynamic spatial dependencies [20].

Traffic flow congestion at intersections of Kathmandu valley was analyzed using f statistical multiplexing and particle swarm optimization [21]. The proposed methodology helped in reducing the average waiting time of vehicles on the considered junctions.

Sakurada et al. [22] modeled an agent-based cyber-physical systems architecture for smart parking systems. The model describes the interconnection between the intelligent agents and the physical parking asset controllers and the system proved to also be adaptable for bicycle parking and car parking.

Over other techniques existing in the literature as alternatives (parallel artificial intelligence, distributed problem solving [23]), multi-agent systems have the advantages of being composed by agents that can interact (in order to distribute the tasks and to plan and monitor the team) and can perform autonomous actions for the specific tasks they are responsible for. For smart cities models, these advantages are essential in order to automate the monitoring processes.

The proposed MAS architecture was designed according to intelligent urban traffic management processes in Birmingham city and the agents were enhanced with intelligence after performing an experimental phase. The experimental results show that the k-nearest neighbor and random tree models forecast the traffic flow in Birmingham city with maximum direction accuracy. Road junction and car parking occupancy rates were predicted with high accuracy by the k-nearest neighbor model. Faults can be detected using decision trees classifiers when high accuracy rates are necessary. When a minimum time building model is more important, then k-nearest neighbor model is recommended. The model that best forecasted and classified the proposed datasets were integrated in MAS. The proposed project has its own communication protocol, improving the communication between agents.

The paper has the following sections: multi-agent system architecture, experiments and results (dataset description, forecasting results, classification results and mass testing) discussion and conclusions.

## 2. Multi-Agent System Architecture

**Figure 1:**
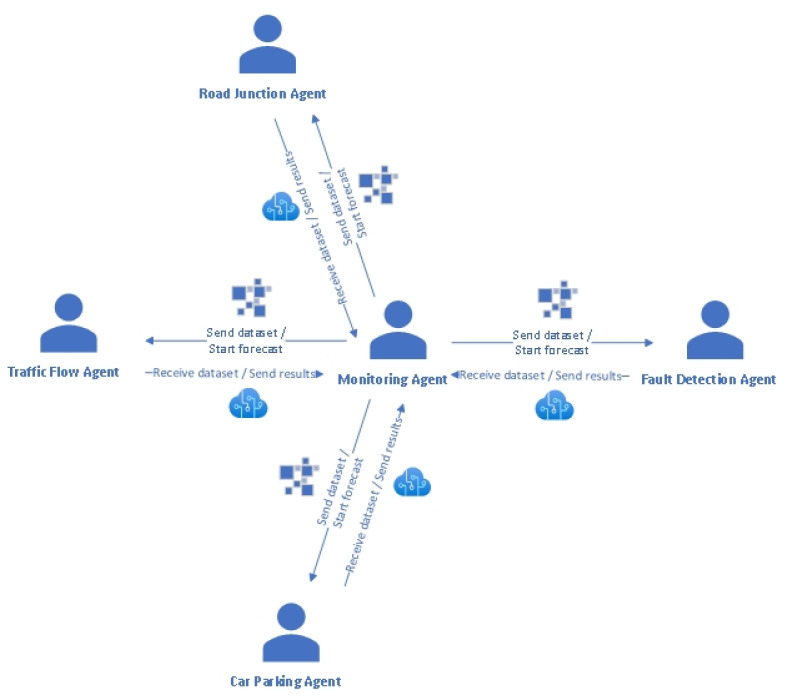
Multi-agent system architecture.

The designed agents have specific goals, ranging from traffic forecasting to fault detection and process monitoring. Figure 1 summarizes the interactions between different agents of the system. The monitoring agent sends the datasets to the forecasting agents as follows: the traffic flow dataset is sent to the traffic flow agent, the road junction dataset is sent to the road junction agent and the car parking dataset is sent to the car parking agent. Together with the dataset, the monitoring agent sends the message “Forecast” to the abovementioned agents. These agents confirm receiving the datasets and the messages from the monitoring agent and inform this agent about starting the forecasting operation. When the training and testing phases are completed, the obtained models, together with the performance measures and forecasted data, are sent back to the monitoring agent. In this way, the urban traffic is monitored and forecasted in real time with an automatic mechanism; therefore, traffic congestion, time spent in road junctions and crowded car parking can be avoided. On the other hand, faults that can occur can be automated, detected and classified by the fault detection agent. This agent receives the fault detection dataset from the monitoring agent together with the classify message. The fault detection agent runs the classification model and predicts the class (type of fault) of the new instance. The discovered model, the performance measures’ values and the class value of the tested instance are sent back to the monitoring agent. This can inform the human expert in real time about the occurrence of a fault in order to solve the problem in time.

**Figure 2:**
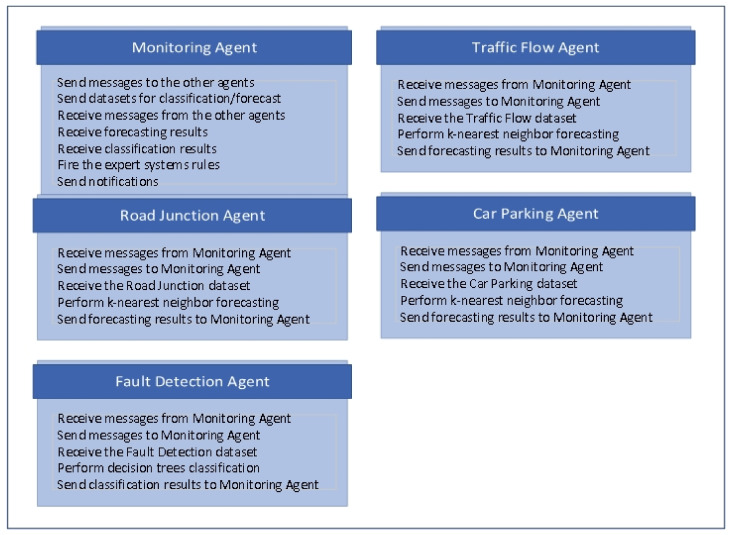
Agents’ behaviors.

Figure 2 describes the behaviors of the designed agents in detail. The traffic flow agent, road junction agent and car parking agent use k-nearest neighbor (IBk implementation) forecasters, and the fault detection agent learns the received dataset using the decision tree (J48 implementation) classification model.

In the proposed agent-based modeling approach, the monitoring agent sends the datasets to be evaluated and receives the forecasting/classification models and results. Other approaches also integrate simulation modeling with machine learning [24,25,26,27].

The communication protocol includes a series of messages and their identifiers (the symbol # encodes the end of a message):

SendDataset = 60 SendDataset#;
ReceiveDataset = 61 ReceiveDataset#;
StartForecast = 62 StartForecast#;
ReceiveForecastResults = 63 ReceiveForecastResults #;
StartClassification = 64 StartClassification #;
ReceiveClassificationResults = 65 ReceiveClassificationResults #;
SendRequest = 70 SendRequest#;
AckRequest = 71 AckRequest#;
WaitingForResponse = 72 WaitingForResponse#;
SendingResponse = 80 SendingResponse#;
AckResponse = 81 AckResponse#;
ConfirmedReceivedResponse = 82 ConfirmedReceivedResponse#;
IdentifyAnomalies = 91 IdentifyAnomalies #;
Error = 100 Error#;
kNearestNeighbour = knn;
decisionTrees = j48;
separator = #;

The designed agents will send only the message identifiers (e.g., 60, 61, and so on) in order to improve the communication time, taking into account that agents send the same messages many times.

The monitoring agent is the decision maker entity in all these cases, being equipped with an expert system that detects when anomalies and faults appear and sends notification messages.

## 3. Experiments and Results

### 3.1. Dataset Description

The MAS uses four real-time traffic datasets published by Birmingham and the West Midlands councils in [28,29]: fault detection dataset (UTMC Faults), traffic flow dataset (UTMC Flow), road junction dataset (UTMC RTEM) and car parking dataset (UTMC Parking). A brief description of the considered datasets is given in Table 1.

### 3.2. Forecasting Results

For performing forecasting and classification operations, the WEKA data mining tool was used [32]. This is open-source software, written in java, and also contains tools for process design and pre-processing and visualization of the analyzed datasets.

In the experimental phase, a set of models was tested in order to choose the one that better fits the data to be learned. The dataset, in attribute relation file format (.arff), was loaded and was sent as input to IBk, KStar and random tree learning methods. IBk and KStar are lazy learning methods, and random tree is a decision tree-based forecaster. Other types of learning methods were also tested, but the obtained results showed that these methods learned the proposed dataset with low accuracy rates.

Table 2 describes the obtained results with the designed forecasting architecture. Direction accuracy and root-mean-square error performance evaluating measures show that all the proposed models are suitable for learning the traffic flow dataset.

Direction accuracy compares the forecast direction (upward or downward) to the actual realized direction [33]. High direction accuracy rates were achieved due to the uniform distribution and repeatability of data for the occupancy attribute.

**Figure 3:**
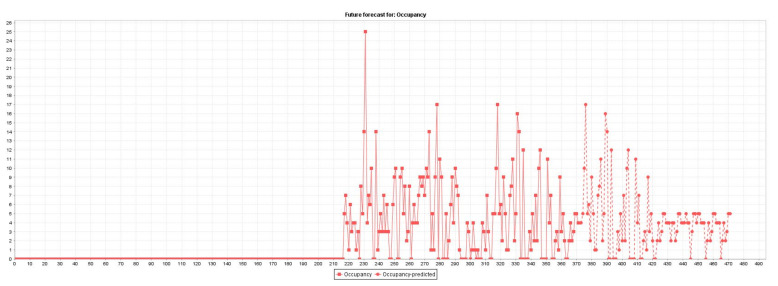
Traffic flow occupancy rates forecasting.

Figure 3 plots the actual (371 instances) and predicted (100 instances) traffic flow occupancy rates.

A similar architecture was also designed for predicting the road junction occupancy rates. Table 3 shows that only the k-nearest neighbor (IBk) model forecasted, with one neighbor (k = 1) returning high direction accuracy and root-mean-square error rates. The performed experiments show that the IBk forecaster is suitable to be implemented in the road junction agent in order to be used on real-time data.

**Figure 4:**
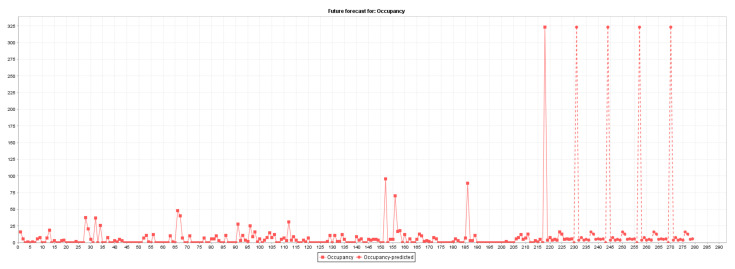
Road junction occupancy rate forecasting.

Figure 4 plots the actual (229 instances) and predicted (100 instances) road junction occupancy rates. Actual values are represented by squares, and the forecasted values are represented by circles.

In car parking occupancy rate forecasting, better results were obtained using random forest compared to the random tree model. However, lazy learners also remained the most suitable forecasters for the car parking dataset (Table 4).

**Figure 5:**
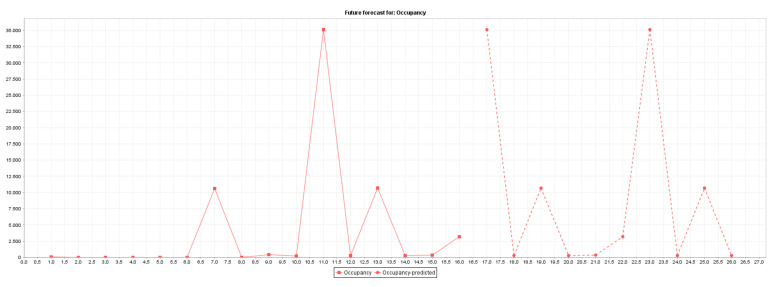
Car park occupancy rate forecasting.

Figure 5 plots the actual (16 instances) and predicted (10 instances) car parking occupancy rates.

Experiments were performed on similar data in a previous study [34], also highlighting the performance of the k-nearest neighbor (IBk) model compared to other models.

Other studies [35,36] have used polynomial fitting, Fourier series, k-means clustering, time series and evolutionary deep learning for car park occupancy prediction in Birmingham.

### 3.3. Classification Results

**Figure 6:**
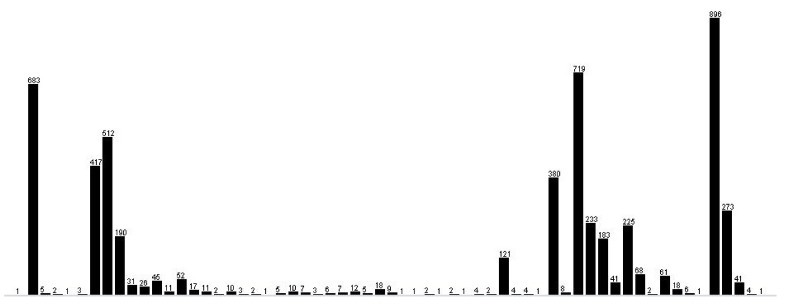
Instances of distribution for class attributes (61 classes).

The employed visualization techniques highlight that the dataset is unbalanced in terms of class instance distribution (Figure 6). Classification methods were also chosen according to this information.

The aim is to build a performant model and to detect the fault type for a new instance, after learning a training file with known classes for its instances (CPU Temperature Fault—temperature is excessive, Intermittent TX fault, Remote attended, Identified lamp fault, CS reply stuck, Identified red lamp fault, Controller synchronization fault, Pelican signals off, Hurry call detected, Invalid stage transition (J)/No vehicle green confirm (P), Lamps off, Signals stuck in intergreen, Pedestrian Confirm/Vehicle Green (PC/GX) reply conflict, and so on). Taking into account the data source (car_park, traffic_signal, meteorological, etc.), data type (CRS ANPR, SIEMENS UTC, Swarco, Cloud Amber, CA Traffic, ANPR) and the other attributes’ values as presented in Table 1, the best models are generated and used for real-time data.

**Figure 7:**
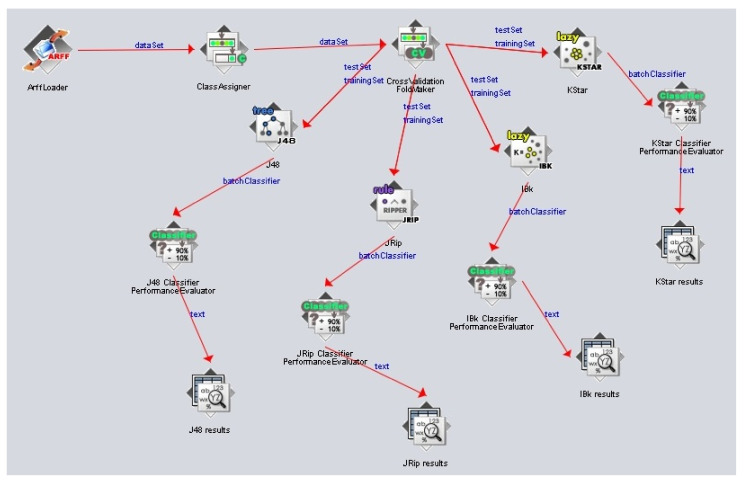
Fault classification process flow.

The knowledge flow (Figure 7) describes the steps of the classification process:

- Loading the fault detection dataset.
- Assigning the class attribute (fault type attribute).
- Splitting the dataset into a training set and testing set. The dataset was split into 10 equal folds (parts): nine folds for training and one fold for testing.
- Building the classifier model: the data were learned using lazy models (IBk, KStar), decision tree models (J48) and decision rules models (JRip).
- Evaluating the discovered model: For this task, a 10-fold cross-validation technique was used. This performs 10 runs, and at each run, it uses a different fold for testing and the remaining nine folds for training. Finally, the average classification accuracy for the 10 runs is computed.
- Presenting the obtained results to the user: for each classifier used, the discovered model, together with the performance measure (classification accuracy, time taken to build model, true positive rates, false negative rates, confusion matrix, etc.) values, is shown.

**Figure 8:**
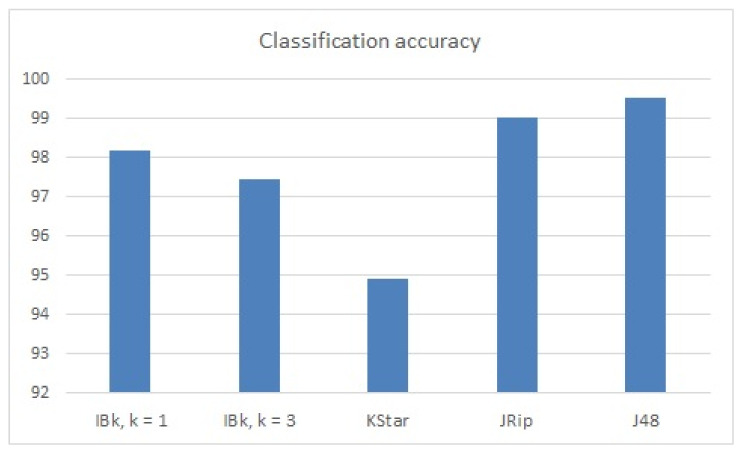
Classification accuracy (fault detection dataset).

**Figure 9:**
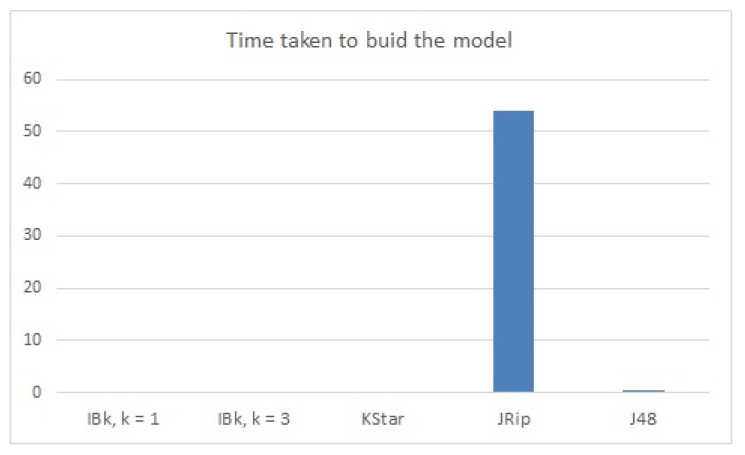
Time taken to build the model (fault detection dataset).

The best classification results in terms of classification accuracy were achieved by the J48 model (99.51%); see Table 5 and Figure 8. The time taken to build this model was also good in terms of a performant model (0.39 s for 5411 learned instances); see Table 5 and Figure 9. Additionally, precision, recall and F-measure reached the highest values using the decision tree model (Table 3).

The building model time was equal to zero when lazy learners were used, but with some decrease in classification accuracy: a 1.36% decrease (comparing to J48) in the case of IBk learning (with 1 neighbor, k = 1), 2.08% decrease when using IBk learning model (with 3 neighbors, k = 3), and 4.62% decrease when learning data with KStar model.

JRip classified the testing instances with high accuracy rates (99.02%) but with an increased building model time (54.01 s).

The best confusion matrix was generated with the J48 model and presents the classification of each class of the dataset. In imbalanced problems, this measure is very important as the classification of underrepresented classes can be evaluated. For instance, for the classification of the “persistent TX fault” class, 1 out of 17 instances was misclassified. Other classes, with very few instances (for instance, the “pelican signals off” class with two instances), which had all the instances assigned to other classes (the “hurry call detected” class), needed a cost matrix (used within the cost-sensitive meta-classifier) with an increased cost for their position (a cost equal to 3000 discovered experimentally) in order to correctly classify all instances. In the case of using a cost matrix to help the classifier better recognize the weakly represented classes, it is necessary to admit a slow drop in the general accuracy rates. In the case presented above, the general accuracy value was equal to 99.42%, equating to a 0.09% drop in general accuracy. It is evident that, even in this case, the J48 model still had the highest accuracy rates compared to other tested classifiers.

### 3.4. MAS Testing

The MAS was implemented in the Java Agent Development (JADE) Framework [37]. This supports communication between agents and uses Foundation for Intelligent Physical Agents and Agent Communication Language (FIPA-ACL language).

**Figure 10:**
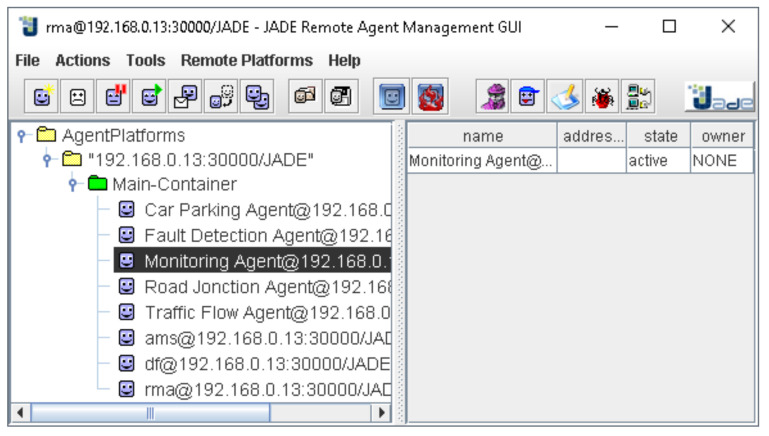
Main container structure.

**Figure 11:**
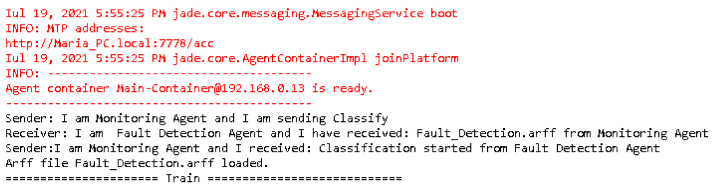
Sample of agents’ exchange of messages.

The designed agents were implemented in the Main JADE Container from the agent platform (Figure 10). All the exchanged messages and performed tasks were shown in the application output (Figure 11). The monitoring agent received the discovered patterns and the predicted data and could perform different actions in order to optimize urban traffic management and control.

The monitoring agent has an embedded expert system implemented with the Jess rule engine [38,39]. The proposed template contains facts for the forecasting unit name, traffic flow occupancy rates, road junction occupancy rates and car parking occupancy rates.

(defglobal ?*maxTrafficFlow* = 12)
(defglobal ?*maxRoadJunction* = 30)
(defglobal ?*maxCarParking* = 10,000)
(deftemplate decision
„Intelligent decision”
(slot name)
(slot traffic_flow (type INTEGER))
(slot road_junction (type INTEGER))
(slot car_parking (type INTEGER)))

In order to check if a forecasted value is anomalous or normal, the monitoring agent fires a set of decision rules:

(defrule checkForecastedValue

„Check forecasted value against maxTrafficFlow”

?p <- (decision {traffic_flow > ?*maxTrafficFlow *})

=>

(add (new Response (str-cat „Forecasted traffic flow occupancy rates greater than maximum global value” ?*maxTrafficFlow* „: „ ?p.name „ = „ ?p.traffic_flow)))

(printout t „Notification: anomalous traffic flow value” ?p.name „ = „ ?p.price crlf))

If the forecasted value exceeds the maximum global value, the monitoring agent decides to send a notification message to the application output, and other management processes can be started.

## 4. Discussion and Conclusions

This paper proposes a multi-agent system to automate urban traffic management and control in Birmingham city. It uses the datasets published by Birmingham and the West Midlands councils in [28,29] and forecasts occupancy rates for traffic flow, road junctions and car parking. The system also detects and classifies the faults that occur in different systems used in the data collection and monitoring processes.

The performed experiments show that the k-nearest neighbor models achieve the best accuracy rates for forecasting the considered time-series, and they have been included in the forecasting agents of the designed system.

In the classification task, the obtained results show that decision trees are suitable for predicting the type of newly occurring faults, considering the classification accuracy and time taken to build the model. This learning technique was used by the fault detection agent in the learning process.

Using the proposed system, important decisions can be made in a shorter time, improving citizens’ lives.

## Figures and Tables

**Table 1:** Dataset description [30].

Dataset	Attribute	Description
traffic flow (371 instances, collected between 2013–2018)	SCN	System code number: a unique value for detector, carpark, etc.
	Description	Road description, e.g., Bristol Road/Oak Tree Lane, Coventry Rd/Kings Rd
	Northing/Easting	OSGB36 datum reference (latitudes and longitudes on the Airy ellipsoid) [31]
	Date	Date and time of instance recording
	Status	Time status, either 0 or 1, indicating false or true
	Occupancy	Detector occupancy rate
	Interval	Numeric, between 0 and 5
	Flow	Flow data produced by vehicle detectors embedded in the road surface per hour
	Speed	Speed data produced by vehicle detectors embedded in the road surface
road junction (229 instances, collected between 2018–2021)	SCN	System code number
	Site/Station	Station ID
	Description	Station description, e.g., A45 Coventry Road/Holder Road, Station 0002
	Northing/Easting	OSGB36 datum reference (latitudes and longitudes on the Airy ellipsoid) [26]
	Date	Date and time of instance recording
	Lane	Numeric, between 0 and 3
	Speed	Speed data produced by vehicle detectors embedded in the road surface
	Headway	The time interval between two vehicles traveling
	Occupancy	Detector occupancy rate
	Vehicles	Number of vehicles at the current timestamp
	Motorbikes	Number of motorbikes at the current timestamp
	Cars	Number of cars at a road junction
	Trailers	Number of trailers at a road junction
	Rigids	Number of rigids at a road junction
	HGVs	Number of HGVs at a road junction
	Buses	Number of buses at a road junction
car parking (16 instances, collected between 2018–2021)	SCN	System Code Number
	Capacity	Car parking capacity
	Disabled	Numeric, with values between 1 and 12
	Description	Car parking description, e.g., BCC Paradise Circus, BCC Town Hall, Broad Street
	Northing/Easting	OSGB36 datum reference (latitudes and longitudes on the Airy ellipsoid) [31]
	Date	Date and time of instance recording
	State	State with the following values: SPACES, OPEN, OTHER
	Occupancy/Percent	Detector occupancy rate
	Trend	With the following values: Other, Static, Filling
	Statistics	String, default 0
	Entry	Number of cars at entry
	Exit	Number of cars at exit
	Queue	Number of cars in queue
fault detection (5411 instances, collected between 2015–2019)	Source	Nominal, with the following values: cctv,car_park, traffic_signal, meteorological, tl, vms, detector, Camera, tl_anpr, tl_scoot, BSI
	SystemCodeNumber	System Code Number
	DataType	Nominal, with the following values: CRS ANPR, SIEMENS UTC, Swarco, Cloud Amber, CA Traffic, ANPR
	SubSystemTypeID	SubSystem ID, numeric
	FaultID	Fault ID, nominal
	FaultText	61 distinct values, e.g., CPU Temperature Fault—temperature is excessive, TX fault—No reply for 3 s
	FaultType	Fault Type, numeric, 47 distinct values
	EquipmentFault	Nominal, with the following values: N, Y, 0, 1
	Communications Fault	Nominal, with the following values: N, Y, 0
	SupplierFault Number	Nominal
	CreationDate	Creation date (timestamp)
	ClearedDate	Cleared date (timestamp)
	LastUpdated	Last updated (timestamp)
	AckTypeId	Numeric: 0, 1

**Table 2:** Traffic flow forecasting results.

Dataset	Forecasting Model	Direction Accuracy	Root Mean Squared Error
Traffic flow	IBk, k = 1	100	0
	KStar	100	0
	Random Tree	100	0

**Table 3:** Road junction forecasting results.

Dataset	Forecasting Model	Direction Accuracy	Root Mean Squared Error
Road junction	IBk, k = 1	100	0
	KStar	85.92	0
	Random Tree	70.29	2.92

**Table 4:** Car parking forecasting results.

Dataset	Forecasting Model	Direction Accuracy	Root Mean Squared Error
Car parking	IBk, k = 1	100	0
	KStar	100	0
	Random Forest	71.47	5127.84

**Table 5:** Classification results.

Dataset	Classification Model	Accuracy (%)	Precision	Recall	F-Measure	Time (Seconds)
Fault detection	IBk, k = 1	98.15	0.981	0.982	0.981	0
	IBk, k = 3	97.43	0.972	0.974	0.973	0
	KStar	94.89	0.952	0.949	0.945	0
	JRip	99.02	0.985	0.990	0.987	54.01
	J48	99.51	0.992	0.995	0.994	0.39

## Data Availability

The data presented in this study are openly available in https://data.birmingham.gov.uk/dataset/wm-utmc (accessed on 17 July 2021) and http://butc.opendata.onl/AL_OpenData (accessed on 17 July 2021).
